# Dissociative experiences of compartmentalization are associated with food addiction symptoms: results from a cross‐sectional report

**DOI:** 10.1007/s40519-023-01555-2

**Published:** 2023-03-03

**Authors:** Giuseppe Alessio Carbone, Elena De Rossi, Elisabeth Prevete, Lorenzo Tarsitani, Ornella Corazza, Chiara Massullo, Benedetto Farina, Massimo Pasquini, Ines Taddei, Massimo Biondi, Claudio Imperatori, Francesco Saverio Bersani

**Affiliations:** 1 Cognitive and Clinical Psychology Laboratory, Department of Human Sciences, European University of Rome, Via degli Aldobrandeschi 190, 00163 Rome, Italy; 2 Department of Human Neurosciences, Sapienza University of Rome, 00185 Rome, Italy; 3 Department of Clinical, Pharmaceutical and Biological Sciences, University of Hertfordshire, Hatfield, AL10 9EU UK; 4 Experimental Psychology Laboratory, Department of Education, Roma Tre University, 00185 Rome, Italy

**Keywords:** Food addiction, Dissociative symptoms, Compartmentalization, Eating disturbances, Psychopathology

## Abstract

**Purpose:**

Studies have shown significant associations of dissociative symptoms with both eating and addictive disorders; however, the different forms of dissociation have been relatively understudied in relation to food addiction (FA). The main aim of this study was to investigate the association of certain forms of dissociative experiences (i.e., absorption, detachment and compartmentalization) with FA symptoms in a nonclinical sample.

**Methods:**

Participants (*N* = 755; 543 women; age range: 18–65; mean age: 28.22 ± 9.99 years) were evaluated using self‐report measures of FA, dissociation, eating disturbances, and general psychopathology.

**Results:**

Compartmentalization experiences (defined as pathological over-segregation of higher mental functions) were independently associated with FA symptoms (*β* = 0.174; *p* = 0.013; CI = [0.008; 0.064]) even when confounding factors were controlled for.

**Conclusion:**

This finding suggests that compartmentalization symptoms can have a role in the conceptualization of FA, with such two phenomena possibly sharing common pathogenic processes.

*Level of evidence*: Level V, cross-sectional descriptive study.

**Supplementary Information:**

The online version contains supplementary material available at 10.1007/s40519-023-01555-2.

## Introduction

Over the last years the construct of Food Addiction (FA) has attracted interest in both clinical and research fields, mainly in relation to certain forms of obesity, to eating disorders (EDs), especially binge-type EDs, and to addictive behaviors [1–3]. To date there is still not an unequivocal definition of FA, and debates have been occurring on whether FA is an independent nosographic condition or not, and whether it should be conceptualized as a form of ED or as a form of addictive disorder [2, 4, 5]. FA has been conceptualized as a behavioral pattern characterized by uncontrolled and dysregulated consumption of hyper-palatable and highly processed foods (i.e., containing refined carbohydrates and/or added fats such as pizza or French-fries) [3, 6, 7], as well as a behavioral pattern with clinical (e.g., continued overuse despite negative consequences) and neurophysiological (e.g., altered dopamine expression) overlaps with addiction, certain forms of obesity, and binge-type EDs [1, 2, 5].

Under a psychopathological point of view, it has been reported in both clinical (e.g., overweight and obese patients seeking weight-loss treatment) and non-clinical (e.g., students, general population) samples that higher FA symptoms are associated with different physical and psychological conditions such as increased craving and impulsivity, higher body mass index (BMI), and altered eating behavior with recurrent binge eating episodes [e.g., 8–12]. FA has also been related with a range of psychiatric disorders, such as depression, anxiety and post-traumatic stress disorder [4, 13].

The etiology of FA and the concomitant features implied in its maintenance are still unclear. For example, although the genetic investigation of FA is still inconclusive [14], it has been recently reported that higher polygenic scores related to dopamine signaling predicted higher FA symptoms in a sample of university students [15]. Also, previous reports [e.g., 16–23] have detected associations between exposure to traumatic experiences, especially when they occur during childhood, and FA severity. In this regard, it is well known [24, 25] that one of the pathogenic processes induced by traumatic experiences is dissociation, which is considered a psychopathological phenomenon frequently detected in both eating and addictive disorders [26–37].

According to the last edition of the Diagnostic and Statistical Manual of Mental Disorders (DSM-5), the term dissociation refers to a “disruption of and/or discontinuity in the normal integration of consciousness, memory, identity, emotion, perception, body representation, motor control, and behavior”; as specified in the same text, “dissociative symptoms can potentially disrupt every area of psychological functioning”, and “dissociative disorders are frequently found in the aftermath of trauma” [38].

Considering the large spectrum of dissociative manifestations, scholars [39, 40] have conceptualized dissociation along a continuum ranging from “normal” (e.g., absorption, "the ability to be totally involved and absorbed in a activity"; [41]), to “pathological” dissociative experiences. It has also been argued that it is unlikely that such heterogeneous dissociative symptoms belong to the same psychopathological category and originate from the same pathogenic process [24, 40, 42–44].

In relation to “pathological” clinically-relevant forms of dissociation, factor analysis investigations, clinical and taxometric studies have suggested the existence of at least two clusters of manifestations [40, 43, 45]. Detachment phenomena are considered as pathological forms of dissociation mainly expressed by symptoms of depersonalization and derealization, reflecting a detachment from the self and reality; compartmentalization phenomena are considered as pathological forms of dissociation which refer to the segregation of integrated functions such as memory or body schema/image, and might be characterized by clinical manifestations including dissociative amnesia or altered emotional and identity control [40].

Assessment and treatment of dissociative manifestations are crucial in clinical practice. Indeed, it is known that the presence of dissociative symptoms can contribute to worsen clinical outcomes and treatment response in several psychiatric disorders, such as EDs [46], borderline personality disorder [47] and obsessive–compulsive disorder [48]. Of relevance, studies have documented the significant association of dissociative symptoms with both eating and addictive disorders [26–37], raising the possibility that certain dysfunctional behaviors (e.g., diminished control over the consumption of food/ substances) might be in part conceptualized as dissociative-like phenomena aimed at facing negative and stressful mental states [33, 49, 50].

Dissociation has however been rarely studied in relation to FA. Thus, the aim of the current investigation is to explore the association of different forms of dissociative experiences with FA symptoms in a nonclinical sample of adults. We hypothesized that pathological dissociation (i.e., detachment and compartmentalization symptoms) is independently and positively associated with FA symptoms, even after controlling for confounding clinical (i.e., EDs-related symptoms, general psychopathology BMI, substance use) and socio-demographic (i.e., age, sex, educational achievement and occupation) variables related to FA [4, 51, 52].

## Materials and methods

### Participants and procedure

In this study 755 participants were enrolled (543 females, mean age: 28.22 ± 9.99 years; age range: 18–65) between September 2020 and May 2021. They voluntarily and anonymously answered a survey shared online, after having provided an informed consent (without receiving any payment or compensation). The following inclusion criteria were used for the current study: (i) age between 18 and 65 years, (ii) good ability to understand written Italian, (iii) correct response to one attentional quality check item, and (iv) the provision of written consent. The choice of performing the study in a nonclinical sample of adults was driven by data suggesting that such study group can offer an opportunity to understand the development and maintenance of a mental health condition within a continuous spectrum of severity [53].

The amount of participants was selected following an a priori power analysis conducted using G*Power 3.1 software [54]. It revealed that, given a probability level of 0.05, a sample size of 550 was required to achieve a small effect size (f^2^ = 0.02) with power = 0.80 in a linear regression analysis with three tested predictor and 14 total number of predictors (details on the predictors are provided below). Since this study is part of a wider research focused on psychopathology and addictions, the sample partially overlaps with that of other studies previously published by our research group [31, 55–57].

### Measures

The following socio-demographic and clinical data were collected for each participant: age, sex, employment, education, substances use, tobacco use, and self-reported weight and height (in order to calculate the BMI). Participants were also asked to answer the following questionnaires: the Dissociative Experience Scale II [DES-II; 58], the modified Yale Food Addiction Scale 2.0 [mYFAS 2.0; 59], the Eating Attitudes Test-26 [EAT-26; 60], the CAGE questionnaire [61], and the Brief Symptom Inventory [BSI; 62]. Socio-demographic and clinical data are listed and described in Table 1.

**Table 1:** Socio-demographic and clinical data of the sample (N = 755)

Age—M ± SD	28.22 ± 9.99
Women—N (%)	543 (71.92)
Occupation	
Unemployed—N (%)	73 (9.67)
Students—N (%)	418 (55.36)
Employed—N (%)	264 (34.97)
Educational achievement	
Primary or secondary school—N (%)	16 (2.12)
College—N (%)	405 (53.64)
Graduation—N (%)	334 (44.24)
Substance use in the last 12 months^a^—N (%)	97 (12.85)
Tobacco use in the last 12 months—N (%)	265 (35.10)
BMI—M ± SD	22.63 ± 3.93
Underweight	71 (9.40)
Normal weight	533 (70.60)
Overweight	117 (15.50)
Obesity	34 (4.50)
DES II—M ± SD	13.62 ± 12.20
Absorption—M ± SD	19.00 ± 14.89
Compartmentalization—M ± SD	5.97 ± 9.97
Detachment—M ± SD	7.09 ± 12.90
DES II > 29—N (%)	71 (9.40)
mYFAS 2.0—M ± SD	1.09 ± 2.05
Food addiction—N (%)	75 (9.93)
EAT-26—M ± SD	10.99 ± 13.38
EAT-26 ≥ 20—N (%)	128 (16.65)
CAGE—M ± SD	0.44 ± 0.88
CAGE ≥ 2—N (%)	94 (12.45)
BSI	
GSI—M ± SD	1.08 ± 0.77
BSI cut-off—N (%)	204 (27.02)

*BMI*  Body Mass Index, *DES II*  Dissociative Experiences Scale II total score, *mYFAS 2.0*  modified Yale Food Addiction Scale 2.0 total score; *EAT-26*  Eating Attitude Test-26 total score, *CAGE*  cut-down/annoyed/guilty/open-eye questionnaire total score, *BSI*  Brief Symptom Inventory, *GSI*  Global Severity Index score of the BSI

^a^Number of individuals who reported that the most frequently used psychoactive substance in the previous year was one of the following: cannabis, cocaine, heroin or other opiates, hallucinogens, amphetamines or other psychostimulants, tranquillizers, other substances different from alcohol, nicotine, caffeine, and hyper caloric food

The DES-II [58, 63] is a 28-items self-report questionnaire measuring several dissociative experiences. Participants rate each item on a 11-points scale ranging from 0 to 100%, indicating the percentage of time the described experience happens in their everyday life. Higher scores suggest more severe dissociative symptoms; a total score of  30 is considered to reflect pathological dissociation [58, 64]. According to Mazzotti et al. [45], DES-II items can assess three different categories of dissociative experiences: absorption, compartmentalization and detachment. In the current study, we used the Italian validated version of the scale [45]. In our study Cronbach’s α was 0.94 for the DES-II total score, 0.91 for the absorption, 0.81 for the compartmentalization, 0.88 for the detachment subscales.

The mYFAS 2.0 [59] is a 13-items scale assessing FA in the previous 12 months according to DSM-5 criteria for Substance-related and Addictive Disorders [38]. While 11 items measure symptoms of FA (e.g., tolerance and craving), 2 items assess FA-related impairment and distress. People are asked to rate each item on an 8-point Likert scale ranging from “never” to “every day”. The scale has two scoring options: continuous or categorical (i.e., diagnostic version). Continuous scores can range from 0 to 11, indicating the number of diagnostic criteria met, whereas FA diagnosis is met when at least 2 symptoms and the presence of eating-related clinical impairment or distress are reported. In our study we used an Italian validated version of the scale [65] and Cronbach’s alpha was 0.91.

The EAT-26 is a 26-item self-report questionnaire investigating ED-related symptoms and concerns [60]. Subjects are asked to rate each item according to a 6-point Likert scale (from “never/rarely/sometimes” = 0 to “always” = 3). The total score ranges from 0 to 78. A cut-off of ≥ 20 is commonly used as the threshold value for clinically significant ED-related pathology [66], with higher scores implying more severe ED-related symptoms. In this study, we used an Italian validated version of the EAT-26 that demonstrated good psychometric properties [67]. In our sample the Cronbach’s alpha was 0.93 for the EAT-26 total score.

The CAGE (“Cut-down/Annoyed/Guilty/Open-Eye”) is a 4-item questionnaire used to detect problematic alcohol use [61]. Total scores range between 0 and 4, with higher scores indicating more severe alcohol use problems. According to previous studies [68], a score of ≥ 2 can be used as a cut-off point for detecting problematic alcohol use. In the present study we used the Italian adaptation of the CAGE [69], and the Cronbach’s α was 0.68.

The BSI [62] consists in 53 items assessing nine symptom dimensions: depression, somatization, obsessive–compulsive, interpersonal sensitivity, anxiety, hostility, phobic anxiety, paranoid ideation, and psychoticism. Each item can be rated on a 5-point Likert scale (0–4). The BSI also provides a global severity index (GSI) measuring the overall psychopathological distress, with higher scores reflecting higher self-report severity of the symptoms. As recommended [62] and according to previous studies [70, 71], we used a cut-off score of T63 on the GSI or in two primary symptom dimensions to detect subjects with clinically-relevant psychopathological distress. We used a previously validated Italian version of the BSI [72]. In our sample, the Cronbach’s alpha for the GSI was 0.97.

### Statistical analysis

Statistical analyses were performed using Statistical Package for the Social Sciences 25 (IBM, Armonk, NY, USA). Data were screened for normality according to Kim et al. [73]: specifically, variables were considered normally distributed if the absolute skew value of the distribution was smaller than 2 or the absolute kurtosis value was smaller than 7. Since BMI, detachment and compartmentalization scales were not normally distributed, the correlations were assessed using Spearman’s rho coefficients.

In order to assess the independent contribution of dissociative symptoms in explaining FA severity, a multiple linear regression analysis with mYFAS total score as the dependent variable and DES-II subscales (i.e., absorption, detachment and compartmentalization) as independent variables was performed. Potential confounding clinical (i.e., EAT-26 total scores, CAGE total scores, GSI score, BMI, substance and tobacco use) and socio-demographic (i.e., age, sex, educational achievement, occupation and student status) variables associated with FA [4, 51, 52] were controlled for. Assumptions on multiple regression model were tested according to Williams et al. [74]. Cook’s distances were also computed. Collinearity was assessed through the statistical factor of tolerance and Variance Inflation Factor (VIF). The associations between variables were reported as standardized beta coefficients (*β*) and their *p*-values.

## Results

According to the cutoff scores of the previously mentioned scales, in our sample 71 subjects (9.40%) showed threshold values compatible with pathological dissociation; 75 participants (9.93%) showed threshold values compatible with FA; 128 participants (16.65%) showed threshold values compatible with ED-related pathology; 94 subjects (12.45%) showed threshold values compatible with problematic alcohol use; 204 (27.02%) participants showed threshold values compatible with clinically relevant psychopathological distress. Further, according to the standard BMI cut scores [75], there were 71 (9.40%) underweight, 533 (70.60%) normal weight, 117 (15.50%) overweight, and 34 (4.50%) obese participants.

FA severity was correlated with all DES-II subscales (absorption: rho = 0.301 *p* < 0.001; compartmentalization: rho = 0.273 *p* < 0.001; detachment: rho = 0.298 *p* < 0.001). The mYFAS 2.0 total score was also positively correlated with self-reported BMI (rho = 0.173 *p* < 0.001), EAT-26 total score (rho = 0.471 *p* < 0.001), CAGE total score (rho = 0.185 *p* < 0.001) and GSI (rho = 0.418 *p* < 0.001). Detailed correlations are reported in Supplementary Table S1.

**Fig. 1:**
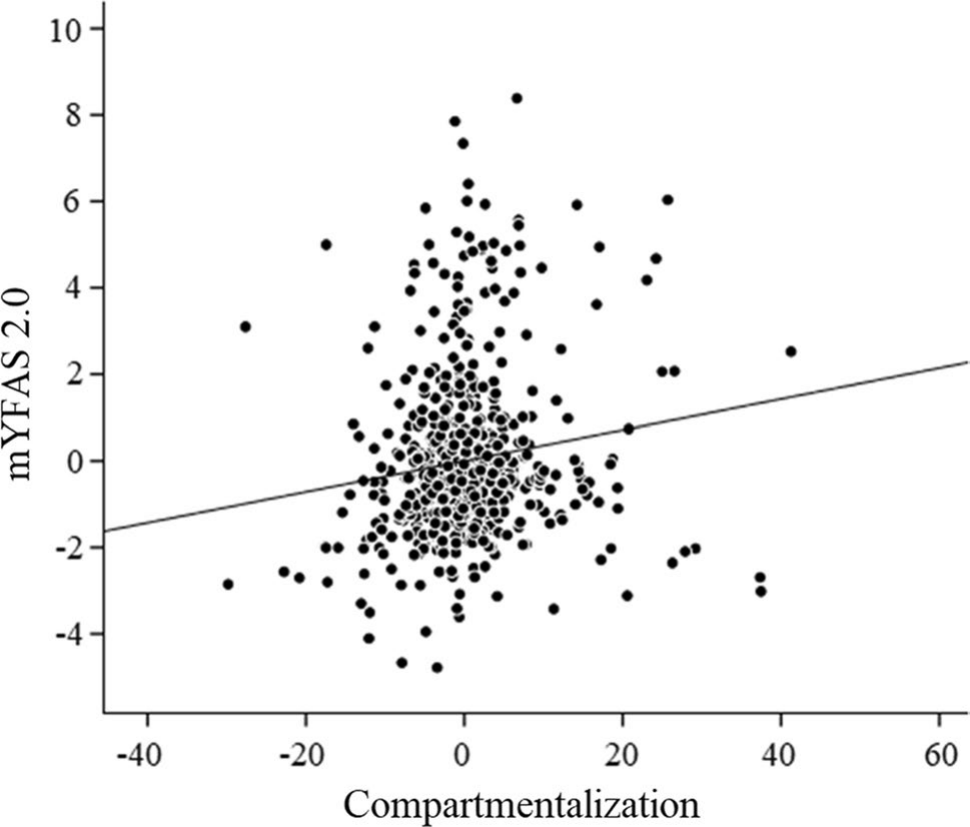
Scatterplot of the association (controlling for confounding variables) between mYFAS 2.0 total score and compartmentalization (*β* = 0.174; *p* = 0.013)

Assumptions on multiple regression were respected with the exception of homoscedasticity. Thus, according to Williams et al. [74], a generalized linear regression model with robust standard errors was performed. The results of the linear regression analysis are reported in Table 2. The model explained 31% of the variability of the mYFAS 2.0 (F_1;753_ = 197.14; *p* < 0.001). Among the different DES-II sub-scales, compartmentalization (*β* = 0.174; *p* = 0.013; CI = [0.008; 0.064]; Fig. 1) was the only DES-II sub-scale independently associated with FA. Education, BMI, EAT-26 and GSI scores were also independently associated with FA scores. No other significant associations were found. The statistical factor of tolerance and VIF showed that there were no interfering interactions between the variables (i.e., tolerance values > 0.10 and VIF < 5). Cook’s distances were also satisfactory (i.e., max value = 0.09).

**Table 2:** Linear regression analysis in all sample (N = 755)

Dependent variable	Adjusted R^2^	F_1;753_	Independent variables	*β*	*p*	[95% CI]
mYFAS 2.0	0.31	197.139^***^	Age	− 0.052	0.219	[− 0.027;0.006]
			Sex	0.012	0.726	[− 0.242;0.347]
			Educational achievement	**0.071**	**0.040**	**[0.012;.529]**
			Employed	− 0.057	0.375	[0.786;0.296]
			Student	− 0.124	0.064	[− 0.1.054;0.030]
			BMI	**0.214**	** < 0.001**	**[0.056;0.167]**
			Illicit drugs use	0.013	0.700	[− 0.329;0.489]
			Tobacco use	− 0.034	0.271	[− 0.405;0.114]
			CAGE	0.066	0.105	[− 0.032;0.338]
			EAT-26	**0.266**	** <0 .001**	**[0.025;0.056]**
			BSI-GSI	**0.287**	** <0 .001**	**[0.508;1.017]**
			Absorption	− 0.065	0.237	[− 0.024;0.006]
			Compartmentalization	**0.174**	**0.013**	**[0.008;0.064]**
			Detachment	0.024	0.709	[− 0.016;0.024]

In bold significant variables associated with mYFAS 2.0 total score

*BMI*  Body Mass Index, *mYFAS 2.0*  modified Yale Food Addiction Scale 2.0 total score, *EAT-26*  Eating Attitude Test-26 total score, *CAGE*  Cut-down/Annoyed/Guilty/Open-eye questionnaire total score, *BSI_GSI*  Global Severity Index of the Brief Symptom Inventory score

Coding system: sex: 1 = man, 2 = woman; employed: 0 = no, 1 = yes, student: 0 = no, 1 = yes; educational achievement: 1 = primary or secondary school, 2 = college 3 = graduation; illicit drugs use: 0 = no, 1 = yes; tobacco use: 0 = no, 1 = yes

^***^p < 0.001

## Discussion

The main aim of the current study was to evaluate the association of different forms of dissociative experiences with FA symptoms in a non-clinical sample of adults, controlling for confounding variables. Our results showed that absorption, compartmentalization and detachment severity were positively associated with FA symptoms. However only compartmentalization remained independently associated with mYFAS 2.0 total scores when confounding variables were taken into consideration, suggesting that this specific dissociative form may have an important role in the conceptualization of FA.

Our results seem in line with previous studies which reported significant associations of dissociative symptoms with both eating and addictive disorders [28, 31, 76, 77], suggesting that certain dysfunctional behaviors (e.g., diminished control over food consumption or substance use) might at least partially be conceptualized as dissociative-like phenomena, which can be aimed at alleviating negative and stressful mental states [26, 33, 49].

To the best of our knowledge, this is the first study investigating the association of specific forms of dissociative experiences (i.e., absorption, detachment and compartmentalization) with FA. According to Holmes et al. [40], compartmentalization is a “deficit in the ability to deliberately control processes or actions that would normally be amenable to such control”. More specifically, compartmentalization is considered as a disturbance of the normal integration of higher mental functions including consciousness, memory and self-identity, as well as executive functions and affective/behavioural regulation [42, 43]. The observed association of compartmentalization severity with FA symptoms might reflect a common pathogenic process, possibly linked to previous traumatic experiences, characterized by poor top-down control and poor self-control, which have been reported in individuals with both high level of FA [78, 79] and dissociative symptoms [80]. Consistently, it is known that both FA symptoms [e.g., 16–22] and compartmentalization symptoms [24, 25, 44] are strongly related to a history of traumatic experiences. More specifically, while detachment symptoms are common even in absence of previous traumatic events [43, 44], compartmentalization symptoms are strongly linked to developmental traumas that typically generate difficulties in the regulation of impulses, emotions, and behavior [25, 44].

On the other hand, the symptoms of compartmentalization concern the sphere of self-identity and body image. Indeed, several authors [e.g., 40, 81, 82] have suggested that compartmentalization experiences also comprise somatoform manifestations of dissociation (i.e., symptoms that phenomenologically involve the body) and that a lack of integration of one’s sense of self might be related to body image distortion [83]. Accordingly, in a non-clinical sample of students, Fuller-Tyszkiewicz and Mussap [84] reported the association between somatic symptoms of dissociation and both body image dissatisfaction and body size distortion. These results were also replicated in patients with EDs [e.g., 37]. Interestingly, although diagnostic criteria for FA do not include body image related symptoms, the association between FA and body image disturbance is well documented [85–88]. Thus, compartmentalization phenomena might also reflect, at least in part, one of the underlying mechanisms this relationship (e.g., the presence of a pathogenic process interfering with integration of different aspects of the self that involves body representation).

Beyond dissociative symptoms, similarly to what observed in previous studies, our results also showed that FA symptoms were independently and positively associated with psychopathology severity and ED-related pathology [13, 51, 89]. Moreover, according to our regression model, FA symptoms were also positively associated with educational level, and such finding is not consistent with previous evidence. For example, Ayaz and colleagues [90] reported a negative association between educational levels and FA symptoms in a sample of Turkish healthy subjects. Such discrepancy may be explained by differences in study designs and methods (e.g., the use of the YFAS vs the mYFAS 2.0) and also by potential cross-cultural differences that have been observed in relation to FA and other addictive eating behaviors [51].

### Strengths and limits

The results of the present study should be considered in light of some limitations. First, participants were recruited from the general population (i.e., nonclinical sample) using self-report measures, and for this reason an objective assessment of traumatic experiences as well as of mental health status was not performed. Therefore, future studies should investigate the relationship between FA symptoms and dissociative experiences also in clinical setting (e.g., overweight and obese patients seeking weight-loss treatment) using heterogeneous methodology (e.g., questionnaires and structured clinical interviews). Furthermore, we used a cross-sectional design. Thus a causal interpretation of the observed significant associations is not possible (longitudinal approaches should be encouraged). Third, a potential selection bias might be present as certain groups of individuals are more represented in the included sample (e.g., students compared to non-students, females compared to males). Similarly, the survey was disseminated online, thus it might have been more accessible to individuals spending more time on the Internet. Despite this, according to the described cut scores, in the present sample, 71 participants (9.40%) showed threshold values compatible with pathological dissociation and 75 individuals (9.93%) showed threshold values compatible with FA. Such values are not far from the prevalence of both conditions detected by other studies focused on non-clinical samples (i.e., FA = 14% [52]; pathological dissociation = 10.9% [45]), confirming their high rate also in the general population.

### What is already known on this subject?

The association between dissociative symptoms with both eating and addictive disorders is well documented. Similarly, it is known that FA is strongly related with psychopathology. Despite this, dissociation has been rarely studied in relation to FA.

### What this study adds?

To the best of our knowledge this is the first study to investigate the association between dissociation severity and FA symptoms.

Given the strong relationship of dissociative manifestations with FA symptoms, our results highlight the need of paying specific attention on these phenomena from preventive, clinical, and research perspectives. Indeed, it has been established that the presence dissociative symptoms in patients with psychiatric disorders is associated with negative treatment outcomes [24], suggesting the relevance to incorporate specific strategies with more conventional therapies. It is also possible that the presence of certain clusters of dissociative symptoms may influence the treatment response to different forms of interventions as well as the disease course even among individuals with similar FA-related disorders (e.g. certain forms of obesity, binge-type EDs, addiction), consistently with evidence highlighting that specific psychopathological dimensions can modulate clinical outcomes within subjects with the same categorical diagnosis [91, 92]. Overall, our results suggest that compartmentalization symptoms can have an important role in the conceptualization of FA and underline the relevance of further studies on the topic.

## Supplementary Information

Below is the link to the electronic supplementary material.

Supplementary file1 (DOCX 16 KB)

## Data Availability

Aggregated data may be available from the corresponding author upon reasonable request.

## References

[1] di Giacomo E, Aliberti F, Pescatore F, Santorelli M, Pessina R, Placenti V, Colmegna F, Clerici M (2022). Disentangling binge eating disorder and food addiction: a systematic review and meta-analysis. Eat Weight Disord.

[2] Vasiliu O (2021). Current Status of evidence for a new diagnosis: food addiction—a literature review. Front Psychiatry.

[3] Gearhardt AN, Schulte EM (2021). Is food addictive? A review of the science. Annu Rev Nutr.

[4] Burrows T, Kay-Lambkin F, Pursey K, Skinner J, Dayas C (2018). Food addiction and associations with mental health symptoms: a systematic review with meta-analysis. J Hum Nutr Diet.

[5] Gordon EL, Ariel-Donges AH, Bauman V, Merlo LJ (2018). What is the evidence for "Food Addiction"? A systematic review. Nutrients.

[6] Schulte EM, Avena NM, Gearhardt AN (2015). Which foods may be addictive? The roles of processing, fat content, and glycemic load. PLoS ONE.

[7] Wiss D (2022). Clinical considerations of ultra-processed food addiction across weight classes: an eating disorder treatment and care perspective. Curr Addict Rep.

[8] Meule A, Hermann T, Kubler A (2015). Food addiction in overweight and obese adolescents seeking weight-loss treatment. Eur Eat Disord Rev.

[9] Pape M, Herpertz S, Schroeder S, Seiferth C, Farber T, Wolstein J, Steins-Loeber S (2021). Food Addiction and Its relationship to weight- and addiction-related psychological parameters in individuals with overweight and obesity. Front Psychol.

[10] Schulte EM, Gearhardt AN (2018). Associations of food addiction in a sample recruited to be nationally representative of the United States. Eur Eat Disord Rev.

[11] Gearhardt AN, Boswell RG, White MA (2014). The association of "food addiction" with disordered eating and body mass index. Eat Behav.

[12] Murphy CM, Stojek MK, MacKillop J (2014). Interrelationships among impulsive personality traits, food addiction, and Body Mass Index. Appetite.

[13] Piccinni A, Bucchi R, Fini C, Vanelli F, Mauri M, Stallone T, Cavallo ED, Claudio C (2021). Food addiction and psychiatric comorbidities: a review of current evidence. Eat Weight Disord.

[14] Cornelis MC, Flint A, Field AE, Kraft P, Han J, Rimm EB, van Dam RM (2016). A genome-wide investigation of food addiction. Obesity (Silver Spring).

[15] Romer AL, Su Kang M, Nikolova YS, Gearhardt AN, Hariri AR (2019). Dopamine genetic risk is related to food addiction and body mass through reduced reward-related ventral striatum activity. Appetite.

[16] Hoover LV, Yu HP, Duval ER, Gearhardt AN (2022). Childhood trauma and food addiction: the role of emotion regulation difficulties and gender differences. Appetite.

[17] Stojek MM, Maples-Keller JL, Dixon HD, Umpierrez GE, Gillespie CF, Michopoulos V (2019). Associations of childhood trauma with food addiction and insulin resistance in African-American women with diabetes mellitus. Appetite.

[18] Imperatori C, Innamorati M, Lamis DA, Farina B, Pompili M, Contardi A, Fabbricatore M (2016). Childhood trauma in obese and overweight women with food addiction and clinical-level of binge eating. Child Abuse Negl.

[19] Legendre M, Sabourin S, Begin C (2022). Maladaptive eating behaviors and childhood trauma: a focus on food addiction. Cureus.

[20] Offer S, Alexander E, Barbara K, Hemmingsson E, Flint SW, Lawrence BJ (2022). The association between childhood trauma and overweight and obesity in young adults: the mediating role of food addiction. Eat Weight Disord.

[21] Mason SM, Flint AJ, Field AE, Austin SB, Rich-Edwards JW (2013). Abuse victimization in childhood or adolescence and risk of food addiction in adult women. Obesity.

[22] Legendre M, Sabourin S, Begin C (2022). Childhood sexual abuse and food addiction severity in a clinical sample of individuals with overweight or obesity. Eat Weight Disord.

[23] Jacques-Tiura AJ, Lanni DJ, Anderson LA, Naar S (2021). Victimization and food addiction symptoms: direct and indirect effects through emotion dysregulation, impulsivity, and loss-of-control eating. Psychol Women Q.

[24] Farina B, Liotti M, Imperatori C (2019). The role of attachment trauma and disintegrative pathogenic processes in the traumatic-dissociative dimension. Front Psychol.

[25] Farina B, Meares R, Dorahy MJ, Gold SN, O’Neil JA (2022). The traumatic disintegration dimension. Dissociation and the dissociative disorders: past, present, future.

[26] La Mela C, Maglietta M, Castellini G, Amoroso L, Lucarelli S (2010). Dissociation in eating disorders: relationship between dissociative experiences and binge-eating episodes. Compr Psychiatry.

[27] Somer E (2019). Trauma, dissociation, and opiate use disorder. Curr Addict Rep.

[28] Guglielmucci F, Monti M, Franzoi IG, Santoro G, Granieri A, Billieux J, Schimmenti A (2019). Dissociation in problematic gaming: a systematic review. Curr Addict Rep.

[29] Craparo G (2011). Internet addiction, dissociation, and alexithymia. Procedia Soc Behav Sci.

[30] Imperatori C, Innamorati M, Bersani FS, Imbimbo F, Pompili M, Contardi A, Farina B (2017). The association among childhood trauma, pathological dissociation and gambling severity in casino gamblers. Clin Psychol Psychother.

[31] Imperatori C, Barchielli B, Corazza O, Carbone GA, Prevete E, Montaldo S, De Rossi E, Massullo C, Tarsitani L, Ferracuti S, Pasquini M, Biondi M, Farina B, Bersani FS (2023). The Relationship Between Childhood Trauma, Pathological Dissociation, and Behavioral Addictions in Young Adults: Findings from a Cross-Sectional Study. J Trauma Dissociat.

[32] Lyssenko L, Schmahl C, Bockhacker L, Vonderlin R, Bohus M, Kleindienst N (2018). Dissociation in psychiatric disorders: a meta-analysis of studies using the dissociative experiences scale. Am J Psychiatry.

[33] Jacobs DF (1988). Evidence for a common dissociative-like reaction among addicts. J Gambl Stud.

[34] Rabito-Alcon MF, Baile JI, Vanderlinden J (2020). Child trauma experiences and dissociative symptoms in women with eating disorders: case-control study. Children (Basel).

[35] Palmisano GL, Innamorati M, Susca G, Traetta D, Sarracino D, Vanderlinden J (2018). Childhood traumatic experiences and dissociative phenomena in eating disorders: level and asociation with the severity of bine eating symptoms. J Trauma Dissociation.

[36] Gleaves DH, Eberenz KP (1995). Correlates of dissociative symptoms among women with eating disorders. J Psychiatr Res.

[37] Longo P, Marzola E, Martini M, Amodeo L, Abbate-Daga G (2022). Anorexia nervosa and somatoform dissociation: a neglected body-centered perspective. J Trauma Dissociation.

[38] American Psychiatric Association: diagnostic and statistical manual of mental disorders: DSM-5. Arlington, VA, American Psychiatric Association, 2013.

[39] Spiegel D, Loewenstein RJ, Lewis-Fernández R, Sar V, Simeon D, Vermetten E, Cardeña E, Dell PF (2011). Dissociative disorders in DSM-5. Depress Anxiety.

[40] Holmes EA, Brown RJ, Mansell W, Fearon RP, Hunter EC, Frasquilho F, Oakley DA (2005). Are there two qualitatively distinct forms of dissociation? A review and some clinical implications. Clin Psychol Rev.

[41] Allen JG, Fultz J, Huntoon J, Brethour JR (2002). Pathological dissociative taxon membership, absorption, and reported childhood trauma in women with trauma-related disorders. J Trauma Dissociation.

[42] Brown RJ (2006). Different types of “dissociation” have different psychological mechanisms. J Trauma Dissociation.

[43] Butler C, Dorahy MJ, Middleton W (2019). The detachment and compartmentalization inventory (DCI): an assessment tool for two potentially distinct forms of dissociation. J Trauma Dissociation.

[44] Steele K, Dorahy MJ, van der Hart O, Dorahy MJ, Gold SN, O’Neil JA (2022). Dissociation versus alterations in consciousness. Related but different concepts. Dissociation and the dissociative disorders: past, present.

[45] Mazzotti E, Farina B, Imperatori C, Mansutti F, Prunetti E, Speranza AM, Barbaranelli C (2016). Is the Dissociative Experiences Scale able to identify detachment and compartmentalization symptoms? Factor structure of the Dissociative Experiences Scale in a large sample of psychiatric and nonpsychiatric subjects. Neuropsychiatr Dis Treat.

[46] La Mela C, Maglietta M, Lucarelli S, Mori S, Sassaroli S (2013). Pretreatment outcome indicators in an eating disorder outpatient group: the effects of self-esteem, personality disorders and dissociation. Compr Psychiatry.

[47] Kleindienst N, Limberger MF, Ebner-Priemer UW, Keibel-Mauchnik J, Dyer A, Berger M, Schmahl C, Bohus M (2011). Dissociation predicts poor response to Dialectial Behavioral Therapy in female patients with Borderline Personality Disorder. J Pers Disord.

[48] Semiz UB, Inanc L, Bezgin CH (2014). Are trauma and dissociation related to treatment resistance in patients with obsessive-compulsive disorder?. Soc Psychiatry Psychiatr Epidemiol.

[49] McCormick J, Delfabbro P, Denson LA (2012). Psychological vulnerability and problem gambling: an application of Durand Jacobs’ general theory of addictions to electronic gaming machine playing in Australia. J Gambl Stud.

[50] Lyubomirsky S, Casper RC, Sousa L (2001). What triggers abnormal eating in bulimic and nonbulimic women?. Psychol Women Q.

[51] Imperatori C, Fabbricatore M, Vumbaca V, Innamorati M, Contardi A, Farina B (2016). Food addiction: definition, measurement and prevalence in healthy subjects and in patients with eating disorders. Riv Psichiatr.

[52] Praxedes DRS, Silva-Junior AE, Macena ML, Oliveira AD, Cardoso KS, Nunes LO, Monteiro MB, Melo ISV, Gearhardt AN, Bueno NB (2022). Prevalence of food addiction determined by the Yale Food Addiction Scale and associated factors: a systematic review with meta-analysis. Eur Eat Disord Rev.

[53] Marques DR, Gomes AA, de Azevedo MHP (2021). Utility of studies in community-based populations. Sleep Vigil.

[54] Faul F, Erdfelder E, Buchner A, Lang A-G (2009). Statistical power analyses using G* Power 3.1: tests for correlation and regression analyses. Behav Res Methods.

[55] Bersani FS, Barchielli B, Ferracuti S, Panno A, Carbone GA, Massullo C, Farina B, Corazza O, Prevete E, Tarsitani L (2021). The association of problematic use of social media and online videogames with aggression is mediated by insomnia severity: a cross-sectional study in a sample of 18-to 24-year-old individuals. Aggress Behav.

[56] Bersani FS, Accinni T, Carbone GA, Corazza O, Panno A, Prevete E, Bernabei L, Massullo C, Burkauskas J, Tarsitani L (2022). Problematic use of the internet mediates the association between reduced mentalization and suicidal ideation: a cross-sectional study in young adults. Healthcare.

[57] Imperatori C, Panno A, Carbone GA, Corazza O, Taddei I, Bernabei L, Massullo C, Prevete E, Tarsitani L, Pasquini M, Farina B, Biondi M, Bersani FS (2022). The association between social media addiction and eating disturbances is mediated by muscle dysmorphia-related symptoms: a cross-sectional study in a sample of young adults. Eat Weight Disord.

[58] Carlson EB, Putnam FW (1993). An update on the dissociative experiences scale. Dissociation.

[59] Schulte EM, Gearhardt AN (2017). Development of the modified Yale food addiction scale version 2.0. Eur Eat Disord Rev.

[60] Ewing JA (1982). The eating attitudes test: psychometric features and clinical correlates. Psychol Med.

[61] Ewing JA (1984). Detecting alcoholism: the CAGE questionnaire. JAMA.

[62] Derogatis LR, Melisaratos N (1983). The Brief Symptom Inventory: an introductory report. Psychol Med.

[63] Bernstein EM, Putnam FW (1986). Development, reliability, and validity of a dissociation scale. J Nerv Ment Dis.

[64] Bernstein E, Putnam F, Espírito-Santo H, Pio-Abreu J (1986). Dissociative experiences scale. Dissociation.

[65] Imperatori C, Fabbricatore M, Lester D, Manzoni GM, Castelnuovo G, Raimondi G, Innamorati M (2019). Psychometric properties of the modified Yale Food Addiction Scale Version 2.0 in an Italian non-clinical sample. Eat Weight Disord.

[66] Garfinkel PE, Newman A (2001). The eating attitudes test: twenty-five years later. Eat Weight Disord.

[67] Dotti A, Lazzari R (1998). Validation and reliability of the Italian EAT-26. Eat Weight Disord.

[68] Dhalla S, Kopec JA (2007). The CAGE questionnaire for alcohol misuse: a review of reliability and validity studies. Clin Invest Med.

[69] Agabio R, Marras P, Gessa GL, Carpiniello B (2007). Alcohol use disorders, and at-risk drinking in patients affected by a mood disorder, in Cagliari, Italy: sensitivity and specificity of different questionnaires. Alcohol Alcohol.

[70] Grassi L, Caruso R, Mitchell AJ, Sabato S, Nanni MG (2018). Screening for emotional disorders in patients with cancer using the Brief Symptom Inventory (BSI) and the BSI-18 versus a standardized psychiatric interview (the World Health Organization Composite International Diagnostic Interview). Cancer.

[71] Grassi L, Mondardini D, Pavanati M, Sighinolfi L, Serra A, Ghinelli F (2001). Suicide probability and psychological morbidity secondary to HIV infection: a control study of HIV-seropositive, hepatitis C virus (HCV)-seropositive and HIV/HCV-seronegative injecting drug users. J Affect Disord.

[72] De Leo D, Frisoni GB, Rozzini R, Trabucchi M (1993). Italian community norms for the Brief Symptom Inventory in the elderly. Br J Clin Psychol.

[73] Kim H-Y (2013). Statistical notes for clinical researchers: assessing normal distribution (2) using skewness and kurtosis. Restor Dent Endod.

[74] Williams MN, Grajales CAG, Kurkiewicz D (2013). Assumptions of multiple regression: correcting two misconceptions. Pract Assess Res Evaluation.

[75] World Health Organization (2018) Body Mass Index—BMI. https://www.who.int/data/gho/data/themes/topics/topic-details/GHO/body-mass-index.

[76] Palmisano GL, Innamorati M, Susca G, Traetta D, Sarracino D, Vanderlinden J (2018). Childhood traumatic experiences and dissociative phenomena in eating disorders: level and association with the severity of binge eating symptoms. J Trauma Dissociation.

[77] Waller G, Babbs M, Wright F, Potterton C, Meyer C, Leung N (2003). Somatoform dissociation in eating-disordered patients. Behav Res Ther.

[78] Franken IH, Nijs IM, Toes A, van der Veen FM (2018). Food addiction is associated with impaired performance monitoring. Biol Psychol.

[79] Meule A, de Zwaan M, Müller A (2017). Attentional and motor impulsivity interactively predict ‘food addiction’in obese individuals. Compr Psychiatry.

[80] Cavicchioli M, Scalabrini A, Northoff G, Mucci C, Ogliari A, Maffei C (2021). Dissociation and emotion regulation strategies: a meta-analytic review. J Psychiatr Res.

[81] Hagenaars MA, van Minnen A, Hoogduin KA (2007). Peritraumatic psychological and somatoform dissociation in predicting PTSD symptoms: a prospective study. J Nerv Ment Dis.

[82] Spitzer C, Barnow S, Freyberger HJ, Grabe HJ (2006). Recent developments in the theory of dissociation. World Psychiatry.

[83] Mussap AJ, Salton N (2006). A 'rubber-hand' illusion reveals a relationship between perceptual body image and unhealthy body change. J Health Psychol.

[84] Fuller-Tyszkiewicz M, Mussap A (2011). Examining the dissociative basis for body image disturbances. Int J Psychol Stud.

[85] Meadows A, Nolan LJ, Higgs S (2017). Self-perceived food addiction: prevalence, predictors, and prognosis. Appetite.

[86] Wu YK, Zimmer C, Munn-Chernoff MA, Baker JH (2020). Association between food addiction and body dissatisfaction among college students: the mediating role of eating expectancies. Eat Behav.

[87] Imperatori C, Innamorati M, Lamis DA, Farina B, Fabbricatore M, Contardi A (2018). Body uneasiness is associated with food addiction symptoms: a cross-sectional study. Eur Eat Disord Rev.

[88] Lacroix E, von Ranson KM (2021). Body image disturbance partially explains eating-related psychosocial impairment in food addiction. Eat Behav.

[89] Oliveira J, Colombarolli MS, Cordas TA (2021). Prevalence and correlates of food addiction: systematic review of studies with the YFAS 2.0. Obes Res Clin Pract.

[90] Ayaz A, Nergiz-Unal R, Dedebayraktar D, Akyol A, Pekcan AG, Besler HT, Buyuktuncer Z (2018). How does food addiction influence dietary intake profile?. PLoS ONE.

[91] Biondi M, Pasquini M, Picardi A (2018). Dimensional psychopathology.

[92] Insel TR (2014). The NIMH Research Domain Criteria (RDoC) Project: precision medicine for psychiatry. Am J Psychiatry.

