# Supplementary material for: Dissociative experiences of compartmentalization are associated with food addiction symptoms: results from a cross‐sectional report
Source: Eat Weight Disord. 2023 Mar 3;28(1):28. doi: 10.1007/s40519-023-01555-2 (PMC9984353; doi:10.1007/s40519-023-01555-2)
Supplement: Supplementary file 1 — Supplementary file1 (DOCX 16 KB) [file 40519_2023_1555_MOESM1_ESM.docx]

**Supplementary Table 1**. Spearman’s rho correlations in all sample (N=755).

|  | 1 | 2 | 3 | 4 | 5 | 6 | 7 | 8 | 9 |
| --- | --- | --- | --- | --- | --- | --- | --- | --- | --- |
| 1. Age | ̶ |  |  |  |  |  |  |  |  |
| 2. BMI | .239^***^ | ̶ |  |  |  |  |  |  |  |
| 3. mYFAS 2.0 | -.055 | .173^**^ | ̶ |  |  |  |  |  |  |
| 4. EAT-26 | -.046 | .060 | .471^**^ | ̶ |  |  |  |  |  |
| 5. CAGE | -.007 | .072^*^ | .185^***^ | .171^***^ | ̶ |  |  |  |  |
| 6. BSI_GSI | -.282^***^ | -.088^*^ | .418^***^ | .407^***^ | .195^***^ | ̶ |  |  |  |
| 7. Absorption | -.204^***^ | -.001 | .301^***^ | .331^***^ | .156^***^ | .509^***^ | ̶ |  |  |
| 8. Compartmentalization | -.050 | .031 | .273^***^ | .256^***^ | .129^***^ | .342^***^ | .626^***^ | ̶ |  |
| 9. Detachment | -.124^**^ | -.044 | .298^***^ | .326^***^ | .162^***^ | .484^***^ | .625^***^ | .508^***^ | ̶ |
| *Abbreviations*: BMI=Body Mass Index; mYFAS 2.0=modified Yale Food Addiction Scale 2.0 total score; EAT-26= Eating Attitude Test-26 total score; CAGE=Cut-down/Annoyed/Guilty/Open-eye questionnaire total score; BSI_GSI=Global Severity Index of the Brief Symptom Inventory score.  **p*<.05; ***p*<.01; ****p*<.001 | | | | | | | | | |
